# Survival outcomes and surgical intervention of small intestinal neuroendocrine tumors: a population based retrospective study

**DOI:** 10.18632/oncotarget.13632

**Published:** 2016-11-26

**Authors:** Lunpo Wu, Jianfei Fu, Li Wan, Jie Pan, Sanchuan Lai, Jing Zhong, Daniel C. Chung, Liangjing Wang

**Affiliations:** ^1^ Department of Gastroenterology, Second Affiliated Hospital of Zhejiang University School of Medicine, Hangzhou, Zhejiang Province, China; ^2^ Institution of Gastroenterology, Zhejiang University, Hangzhou, Zhejiang Province, China; ^3^ Department of Oncology, Zhejiang University Jinhua Hospital, Jinhua, Zhejiang Province, China; ^4^ Department of Endocrinology, Second Affiliated Hospital of Zhejiang University School of Medicine, Hangzhou, Zhejiang Province, China; ^5^ Department of Gastroenterology, Sir Run Run Shaw Hospital, Zhejiang University, Hangzhou, Zhejiang Province, China; ^6^ Gastrointestinal Unit, Massachusetts General Hospital, Harvard Medical School, Boston, Massachusetts, USA

**Keywords:** small intestine, neuroendocrine tumors, prognostic factors, surgical management, SEER

## Abstract

**Background:**

Small intestinal neuroendocrine tumors (SiNETs) without distant metastasis typically behave in an indolent manner, but there can be heterogeneity. We aimed to define the survival outcomes and impacts of surgical intervention.

**Methods:**

A retrospective cohort study was conducted by using data from the Surveillance, Epidemiology, and End Results (SEER) database. Clinicopathologic features were analyzed in 4407 patients between 2000 and 2012. The cancer specific survival (CSS) was calculated by the Kaplan-Meier method. Multivariable Cox regression models with hazard ratios (HRs) were constructed to analyze survival outcomes and risk factors.

**Results:**

The adjusted incidence of early SiNETs is 1.3/100,000. Tumors are most commonly located in the ileum and are small (≤ 2 cm). The 5-year and 10-year CSS rates were 95.0% and 88.5%, respectively. Age > 50 years, large tumor size (> 2cm), poor differentiation, advanced T classification, and absence of surgical treatment were independent predictors of poor survival. Stratified analysis indicated that surgery significantly improved survival in patients that were white (HR, 0.45), > 50 years old (HR, 0.61), had duodenal tumors (HR, 0.43), large tumors (> 2cm) (HR, 0.32), advanced T classification (T3: HR, 0.29; T4: HR, 0.18) or well differentiation (HR, 0.55). There was no significant survival difference between local resection and radical resection (*P* =0.884).

**Conclusions:**

Early SiNETs have a favorable prognosis. Surgical resection may improve outcomes, particularly in older patients and those with large tumors. More aggressive resections couldn't improve outcomes.

## INTRODUCTION

Neuroendocrine tumors (NETs) are heterogeneous, slow-growing tumors that derive from diffuse neuroendocrine cells throughout the body [1]. Oberndorfer *et al* first described these tumors in the small intestine and coined the term *Karzinoid* (carcinoid) in 1907 [2]. He initially considered carcinoid tumors as benign and “carcinoma-like” before their malignancy was further recognized[3]. In 2010, the World Health Organization (WHO) classification replaced “carcinoid” with the terms neuroendocrine tumors and neuroendocrine carcinomas[4].

The incidence of NETs has increased significantly over the past decades [5–8], partially due to the increased use of endoscopic and cross-sectional image techniques as well as the improved recognition of neuroendocrine histology [9]. A shift in the anatomic location of NETs has also been recognized. The small intestine appears to be the most frequent site, replacing the appendix [10–12]. In addition, SiNETs are the most common small bowel neoplasms, and they account for approximately one third of all neuroendocrine tumors [9, 14–17]. There has also been an increasing percentage of tumors diagnosed at an early stage and a concurrent decrease in patients diagnosed with distant metastases [13].

Several studies have described epidemiological features including race, sex, primary tumor site distribution and survival time in patients with SiNETs in the United States, the Netherlands, and the United Kingdom [6, 14–16]. 60-80% of SiNETs present as localized disease [5]. SiNETs without distant metastases typically behave in an indolent manner, but the specific clinicopathologic features and risk factors associated with survival are largely undetermined. In particular, although surgical resection of the primary tumorand associated mesenteric lymph nodes (LNs) is recommended [17, 18], the impact on survival remains controversial [19, 20]. Some have reported that surgical resection of primary tumor is not associated with improved survival [19]. In addition, the optimal extent of resection in SiNETs is unclear. Despite advances in the understanding and management of SiNETs, the survival rates have remained largely unchanged over the past 40 years [21–23]. Using a large, nationwide, population-based database, we performed a retrospective analysis of early SiNETs to define survival outcomes and the impact of surgical intervention.

## RESULTS

### Clinicopathologic characteristics

**Figure 1:**
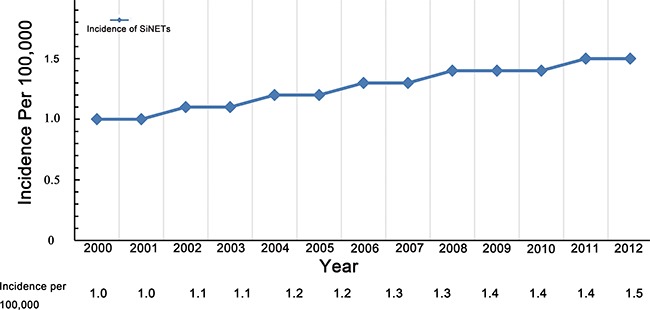
Trends of age-adjusted incidence of SiNETs, Surveillance, Epidemiology, and End Results registry 2000 to 2012 The incidence is presented as the number of tumors per 100,000 (with 95% CIs) age-adjusted for the US standard population. SiNETs, small intestinal neuroendocrine tumours; CI, confidence interval.

A total of 4407 eligible patients were identified from the SEER database. This comprised 70% (4407/6480) of the total number of SiNETs. The adjusted disease incidence is approximately 1.3/100,000 in the population, and an increased incidence of early SiNETs between 2000 and 2012 was observed (Figure 1). The 5-year and 10-year CSS were 95.0% and 88.5%, respectively. The median age of patients was 60 years (IQR 52-69 years). Most patients were older than 50 years (n=3441, 78.1%). The male to female ratio was 51.9:48.1. 3908 (88.7%) patients underwent surgery. Tumors more commonly originated in the ileum (39.9%) and duodenum (32.2%), followed by jejunum (5.2%) and NOS (no otherwise specific) (22.7%). 50.8% were diagnosed as T1 or T2 tumors and 49.8% were classified as N0. 57.9% of tumors were small in size (diameter ≤2 cm) and 89% of early SiNETs were well-differentiated. Histological subtypes mainly consisted of carcinoid (n=3588, 81.4%) and neuroendocrine carcinoma (n=762, 17.3%). Less common subtypes included neuroendocrine adenocarcinoid, enterochromaffin, goblet, atypical, gastinoma and composite histology, and each numbered less than 10. The detailed demographics and clinical characteristics of all SiNETs are listed in Table 1.

**Table 1:** Baseline characteristics and univariate analysis of 4407 patients with localized small intestinal neuroendocrine tumors

Risk Factors	N (%)	5-year CSS (%)	10-year CSS (%)	P value ^a^
Age				<0.001
≤50yrs	966(21.9)	97.3	93.0	
>50yrs	3441(78.1)	94.3	87.0	
Race				0.227
White	3430(77.8)	94.9	87.3	
Black	790(17.9)	94.9	93.8	
Others	136(3.1)	94.7	94.7	
Unknown	51(1.2)	92.3	92.3	
Gender				0.306
Male	2286 (51.9)	96.1	91.0	
Female	2121(48.1)	96.0	88.8	
Marital Status				0.470
Married	2674(60.4)	95.6	88.3	
Unmarried	1432(32.5)	94.1	89.0	
Unknown	301(6.8)	93.9	89.2	
Tumor Location				<0.001
Duodenum	1419 (32.2)	96.4	94.3	
Jejunum	230 (5.2)	93.7	88.1	
Ileum	1759(39.9)	95.7	87.9	
NOS	999(22.7)	92.2	82.2	
Histological type				<0.001
Carcinoid	3588(81.4)	95.7	89.2	
Neuroendocrine	762(17.3)	91.7	86.9	
Other subtypes ^b^	57(1.3)	82.3	- ^e^	
Differentiated grade				<0.001
Well differentiated	3922(89.0)	96.2	89.9	
Moderately differentiated	309(7.0)	89.8	74.9	
Poorly/undifferentiated^c^	146(3.3)	74.9	71.2	
Unknown	30(0.7)	93.3	81.7	
Tumor Size ^d^				<0.001
≤2cm	2553(57.9)	96.9	93.0	
>2cm	951(21.6)	93.0	81.2	
Unknown	903(20.5)	92.0	85.2	
T-classification				<0.001
1	1575(35.7)	97.4	96.7	
2	667(15.1)	98.8	95.4	
3	1426(32.4)	93.4	82.9	
4	454(10.3)	90.2	75.4	
Unknown	285(6.5)	89.1	84.8	
N-classification				0.011
N0	2193(49.8)	96.2	91.2	
N1	1926(43.7)	94.3	85.4	
Unknown	288(6.5)	91.8	86.6	
Surgery				0.001
none	499(11.3)	91.7	82.6	
Surgical resected	3908(88.7)	95.4	89.2	

Abbreviations: CSS = cancer-specific survival; NOS = not otherwise specified.

^a^ Univariate analysis was calculated by the Kaplan-Meier method with the Log-rank test, P value of <0.05 was considered as statistically significant.

^b^ Other subtypes: neuroendocrine adenocarcinoid, enterochromaffin, goblet, atypical, gastinoma and composite histology.

^c^ Poorly differentiated grade and undifferentiated grade were combined as poor/undifferentiated group because of their limited sample sizes;

^d^ Diameter <=2cm.

^e^ The follow-up time is less than 120 months.

### Predictive factors of survival in patients with early SiNETs

We observed that young age (≤50 years), small tumor size (≤2cm), location in duodenum, T1-T2 depth of invasion, N0 classification, and prior surgical resection were associated with better outcomes based on univariate analysis. Race, gender, marital status or histological type were not predictive of outcome (Table 1). Multivariate Cox analysis demonstrated that age>50 years (HR, 1.88; 95%CI, 1.22-2.88), large tumor size (>2cm) (HR, 1.49; 95%CI, 1.01-2.16), poor differentiation (moderately differentiated: HR, 3.33; 95%CI, 2.16-5.14; poorly/undifferentiated grade: HR, 4.98, 95%CI, 3.13-7.92), T3-4 classification (T3: HR, 3.70; 95%CI, 2.09-6.48; T4: HR, 5.21; 95%CI, 2.90-9.36), and absence of prior surgical treatment (HR, 1.99; 95%CI, 1.12-3.56) were significantly associated with lower CSS. Tumor size, location, and N-stage were not independent predictors in proportional hazards analyses (Table 2).

**Table 2:** Multivariate analysis of cancer-specific survival for 4407 patients with localized small intestinal neuroendocrine tumors

Variable(reference)	HR	95.0% CI	P value
Age			
≤50yrs	1	-	-
>50yrs	1.88	1.22-2.88	0.004
Tumor Location	-	-	-
Duodenum	1	-	-
Jejunum	0.93	045-1.94	0.852
Ileum	0.80	0.47-1.34	0.391
Tumor size ^b^	-	-	-
≤2cm	1	-	-
>2cm	1.48	1.01-2.16	0.045
Differentiated grade	-	-	-
Well differentiated	1	-	-
Moderately differentiated	3.33	2.16-5.14	<0.001
Poorly/undifferentiated	4.98	3.13-7.92	<0.001
T-classification	-	-	-
T1	1	-	-
T2	1.06	0.50-2.24	0.883
T3	3.70	2.09-6.48	<0.001
T4	5.21	2.90-9.36	<0.001
N-classification	-	-	-
N0	1	-	-
N1	0.94	0.65-1.35	0.935
Surgery	-	-	-
Without surgery	1	-	-
Surgical resected	0.50	0.28-0.90	0.019

Abbreviations: HR = hazard ratio; CI = confidence interval.

^a^ Multivariate analysis was calculated by the Cox proportional hazards regression model, *P* value of <0.05 was considered as statistically significant.

^b^ Diameter <=2cm.

### Surgical outcomes in SiNET patients

**Figure 2:**
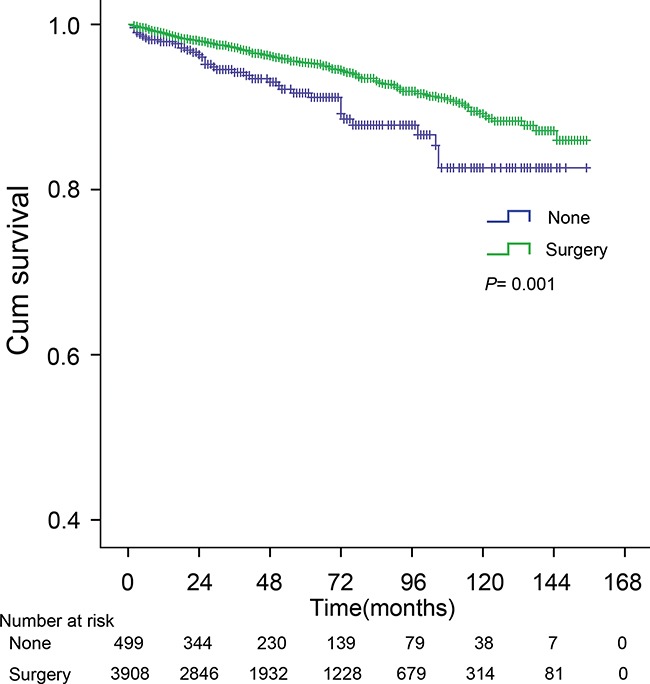
The cancer-specific survival (CSS) curve of patients treated with surgical resection and without surgery The 5-, 10-year CSS for patients treated with and without surgery were 95.4%, 89.2% and 91.7%, 82.6%, respectively (*P* =0.001). *P* value from log-rank test.

**Figure 3:**
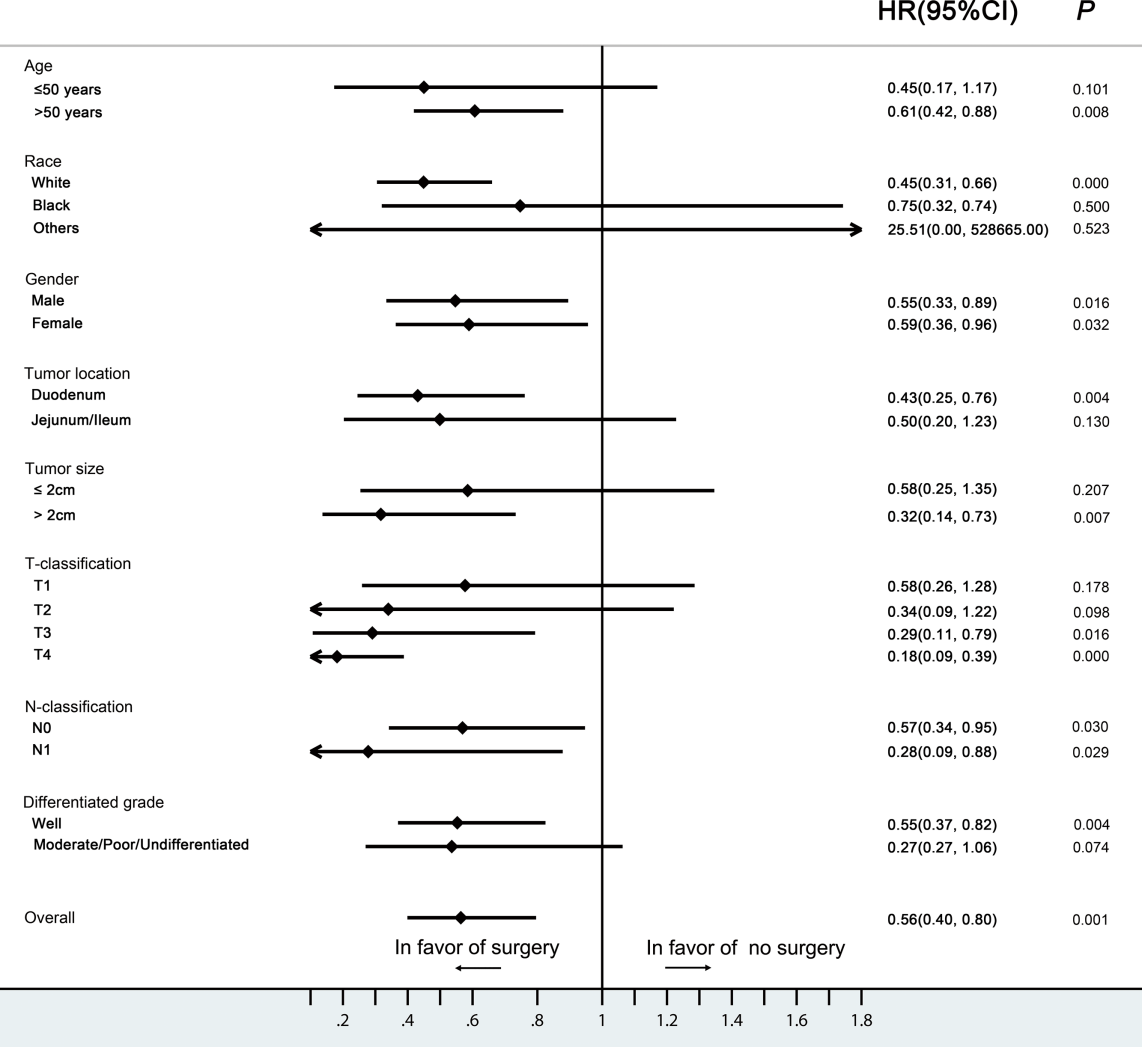
Hazard ratios and 95% CIs in different subgroups of surgery and non-surgery (Forest plot analysis) CI, confidence interval; HR, hazard ratio.

The 5- and 10-year CSS rates for SiNET patients with early disease who underwent surgery were 95.4% and 89.2%, and were 91.7% and 82.6% for those who did not undergo surgery (*P* =0.001) (Figure 2). Further stratified analysis showed that surgery significantly improved the survival in patients that were white (HR, 0.45; 95%CI, 0.31-0.66), >50 years old (HR, 0.61; 95%CI, 0.42-0.88), had a tumor located in the duodenum (HR, 0.43; 95%CI, 0.25-0.76), a large tumor ( >2cm) (HR, 0.32; 95%CI, 0.14-0.73), a T3-T4 tumor (T3: HR, 0.29; 95%CI, 0.11-0.79; T4: HR, 0.18; 95%CI, 0.09-0.39) or well-differentiated histology (HR, 0.55; 95%CI, 0.37-0.83), whereas patients of young age (≤50 years old), with small tumors (≤2cm),T1-T2 classification or moderate/poor/undifferentiated histologic grade did not benefit from surgical management (Figure 3).

### Survival differences between local and radical resection

**Figure 4:**
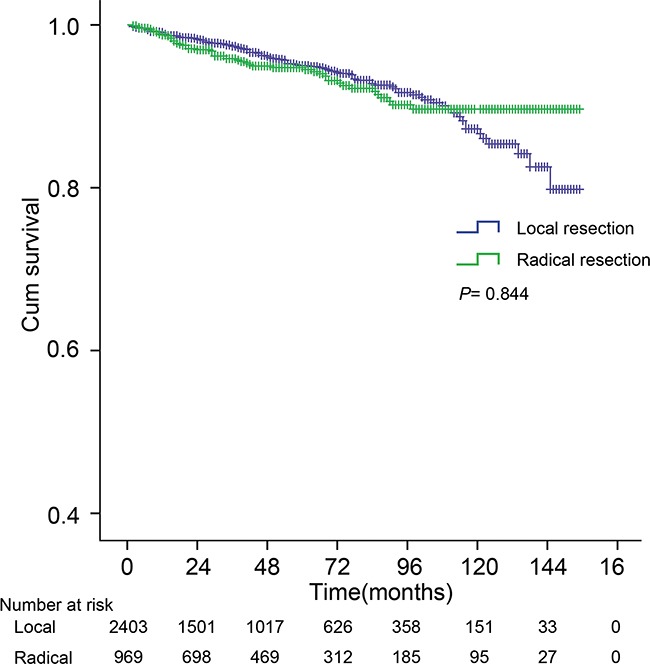
The cancer-specific survival (CSS) curve of patients with local resection (LR) and radical resection (RR) The 5-, 10-year CSS were 95.0%, 87.2% and 94.7%, 89.6%, for LR and RR, respectively (*P* =0.844). *P* value from log-rank test.

**Figure 5:**
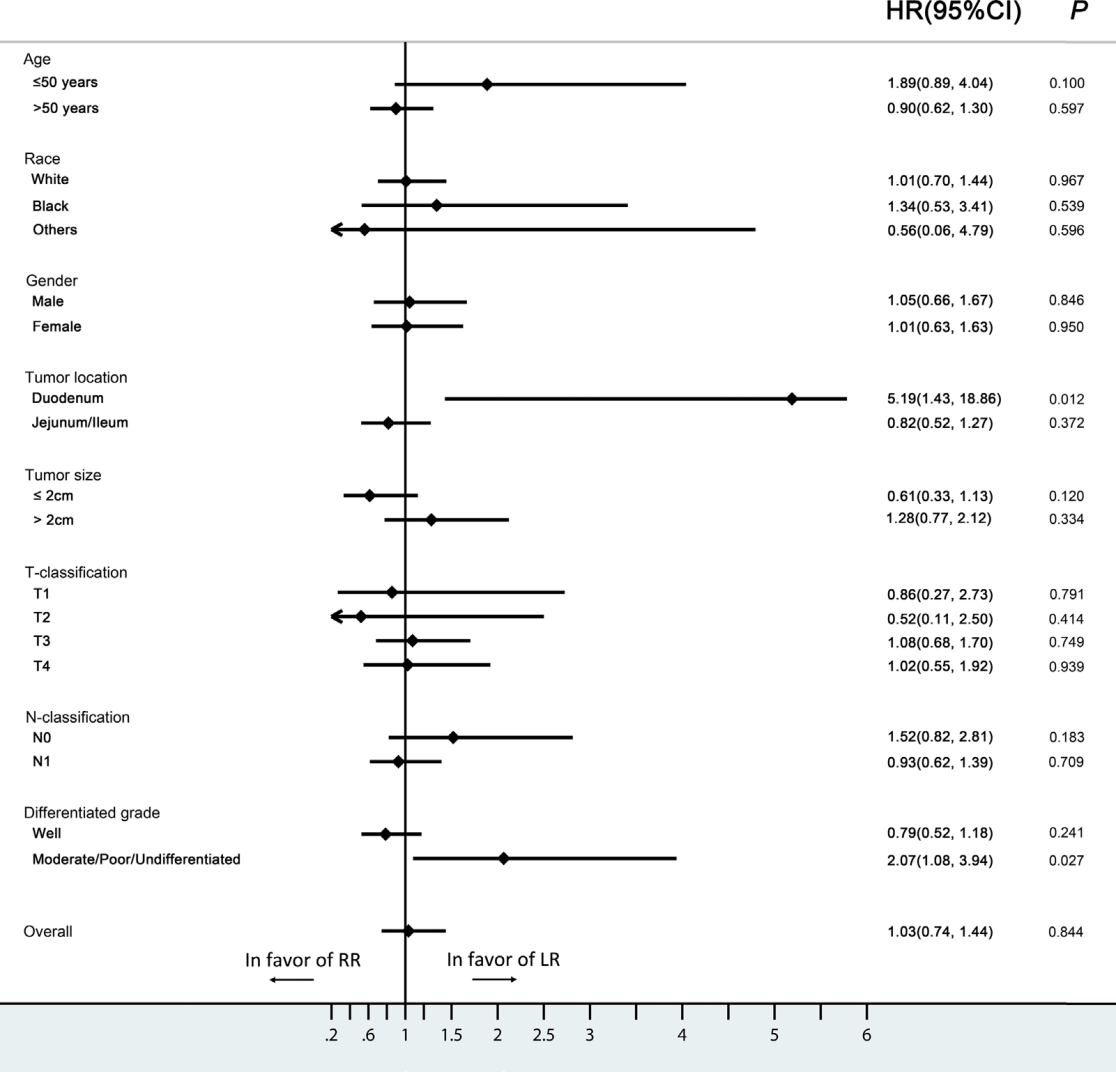
Hazard ratios and 95% CIs in different subgroups of LR and RR (Forest plot analysis) CI, confidence interval; HR, hazard ratio; LR, local resection; RR, radical resection.

Among surgical subgroups (n=3908), Local resection (LR) and Radical resection (RR) were performed on 2403(54.4%) and 969 (22.0%) patients respectively. The 5- and 10-year CSS in patients receiving LR were 95.0% and 87.2%, and were similar (94.7% and 89.6%) in those receiving RR (*P* =0.844) (Figure 4). No clinicopathologic feature was associated with any benefit of RR or LR (Figure 5).

We further analyzed the prognostic effect of the number of lymph nodes (LNs) resected in SiNET patients. 8 LNs was used as the cutoff value because resection of >8 LNs has been associated with an improved survival in patients with SiNETs [24]. No improvement in extended resections with >8 LNs resected was observed in patients with SiNETs(*P* =0.120) (Supplementary Figure S1). Overall, these results indicated that RR is not associated with improved outcomes in SiNET patients.

## DISCUSSION

In this study, we investigated the clinical characteristics and survival outcomes of patients with early SiNETs from 2000 to 2012 from the SEER database. The adjusted incidence is nearly 1.3/100,000 in the population, and this has been increasing over time, consistent with previous studies of SiNETs [13, 25]. Localized or regional SiNETs were generally small in size (≤2cm), more common in older patients (>50 years old) and mostly located in the ileum. There was an equal sex distribution in our analysis, though SiNETs were previously reported to be more common in men [23]. The populations of SiNETs in our study showed a favorable prognosis with a 5-year CSS rate of 95.0%. According to previous studies, the 5-year overall survival rate of SiNETs was approximately 60-70%, which has changed little over the past decades [1, 23], and compares favorably to lower survival rates of 32.5% for adenocarcinomas, 39.9% for stromal tumors, and 49.6% for lymphomas in the small intestine [23]. Patients with early SiNETs displayed more prolonged survival, with 5-year overall survival rates of 70-100% [26]. The high rate of survival observed in this study is potentially explained by inclusion of only non-metastatic SiNETs and using CSS as the endpoint event. Age, tumor size, T classification, and surgical treatment were independent prognostic factors in multivariate analysis. Young age was a powerful positive predictor of survival, consistent with previous reports [23, 27]. However, our analysis did not reveal any statistical differences in race, gender or tumor location, which have been shown to be predictors associated with higher hazard of death in NET in previous studies [5, 10, 23, 27–29].

The most recent North American Neuroendocrine Tumor Society (NANETS) [17] and European Neuroendocrine Tumor Society (ENETS) [18] guidelines for management of localized SiNETs recommend performing resection of primary site with LN removal whenever possible (Level of evidence 4, Grade C) [30]. However, the benefit of surgery is still controversial [19, 20, 31]. Given the rarity of SiNETs, it is impossible to accumulate an adequate number of patients from a single center. Although improved survival can be observed in patients who undergo resection of the primary tumor [32–35], our study revealed that large tumor size (>2cm), white race, older age (>50 years old), location in duodenum, advanced T classification (T3-T4) and poor differentiation might be associated with greater benefit from surgery in SiNETs.

There are currently no specific consensus guidelines on the choice of LR or RR in patients with non-metastatic SiNETs. Burke *et al*. suggested that endoscopic resection may be appropriate for small duodenal tumors (less than 1 cm) [36]. Zyromski *et al*. reviewed 27 patients with primary NETs of the duodenum and concluded that local excision was appropriate for duodenal NETs less than 2 cm in diameter [37]. For patients with localized jejunal or ileal NETs, radical surgery should be recommended [29]. These proposals above were based on the notion that tumor size is a reliable predictor of metastatic spread in duodenal disease and there were no observed metastases from tumors less than 1 cm [36]. For disease in the jejunum and ileum, tumor size does not correspond to the metastatic propensity or liver metastasis can occur even in patients with small primary tumors [29, 38]. Interestingly, in our analysis, no significant influence on survival was found between LR and RR subgroups. Further subgroup analysis revealed that no factors was found to support RR, which is inconsistent with earlier reports [29, 36–38]. Previous studies of SiNETs also showed that laparoscopic approach did have comparable long-term survival compared with open surgery [39]. There may be several reasons to explain the limited benefit of RR. First, multiple synchronous SiNETs are present in up to 30% of SiNETs patients, making any surgical resection unlikely to be complete [26, 40]. Second, RR may unavoidably result in some complications such as ‘short gut syndrome’ that further compromises the patient's quality of life and may even influence survival. Third, when considering the “indolent” nature of the disease in many cases, the effect of any type of surgery may become limited. Finally, although resection of >8 LNs has been associated with a better survival in patients with SiNETs [24], our analysis revealed that expanded lymph node resection did not improve cancer-specific survival. This was similar to a previous study [31] which revealed lymph node metastasis did not correlate with recurrence in duodenal NET.

Our study has several limitations. First, the SEER database reports all incident cancer cases within specified geographic locations but not worldwide. Second, SEER does not provide complete information about the operative approaches, including whether they were endoscopic, laparoscopic or open. We have classified surgery into LR and RR according to the SEER coding system. The procedure of LR was defined as simple resection or partial removal of lesion by polypectomy, excisional biopsy and excision of lesions. RR was defined as debulking or radical resection with an en bloc resection (partial or total removal) [41]. Further clinical studies are required to validate the favorable effect of LR as well as the precise extent of surgical resection. Finally, information about pre- or post-operative therapies and co-morbidities which may affect survival and prognosis are lacking.

In summary, this is the first large population-based study focused on early SiNETs. Our study adds to the current knowledge by defining prognostic factors for survival and the impact of surgical management in localized or regional disease. Surgical resection is recommended, particularly for large tumors and older patients. There was no significant survival difference between LR and RR. Understanding the features that predict outcome in this increasingly common disease is critical.

## MATERIALS AND METHODS

### Data collection

The Surveillance, Epidemiology, and End Results (SEER) program, an authoritative American cancer information database, was initiated in 1973 by collecting information on cancer incidence and survival. The current SEER database collects and publishes cancer data from 18 population-based cancer registries among 14 states across the United States that represent approximately 30% of the population of the United States. SEER coverage includes 26% of African Americans, 38% of Hispanics, 44% of American Indians and Alaska Natives, 50% of Asians, and 67% of Hawaiian/Pacific Islanders. The SEER data contain no identifiers, and is publicly available for studies of cancer based epidemiology and health policy (http://seer.cancer.gov/). SEER database are collected and released annually, reflecting the latest updated information. We received permission to access the research data (Reference Number: 10911-Nov2014). SEER. Stat software was utilized to identify patients from January 2000 to December 2012. The endpoint date of follow-up was Nov. 2014, with a median follow-up of 46 months (range from 2 to 155 months). Patients diagnosed after 2012 were excluded to ensure adequate duration of follow-up. The year and age at diagnosis, gender, race, site record, histological type, differentiated grade, AJCC 7^th^ TNM T-stage, number of metastatic lymph nodes, number of regional lymph nodes examined, survival months and cause of death were retrieved from the SEER database. In an effort to protect patient identity and follow the SEER data use agreement, all data with 10 or less patients are reported as “less than 10”.

### Patient enrollment

The specific inclusion criteria for SiNETs were as follows: (1) the year of diagnosis from 2000 to 2012; (2) site record ICD-O-3 was limited to small intestine (C170-173;C178-179); (3) histological type ICD-O-3 was limited to 8240(carcinoid), 8241 (enterochromaffin cell carcinoid), 8242 (enterochromaffin-like cell tumors), 8243 (goblet cell carcinoid), 8244 (composite carcinoid), 8245 (adenocarcinoid), 8246 (neuroendocrine carcinoma), 8249 (atypical carcinoid), 8013(large-cell neuroendocrine carcinoma), 8041 (small cell neuroendocrine carcinoma), 8152 (Glucagonoma), 8153 (gastrinoma), 8156 (somatostatinoma); (4) SEER stage system was limited to localized or regional. A localized NET was defined as an invasive neoplasm confined entirely to the organ of origin. A regional NET was defined as a neoplasm that extended beyond the limits of the organ of origin directly into surrounding organs or tissue, involved regional lymph nodes, or fulfilled both of the aforementioned criteria [5].

**Figure 6:**
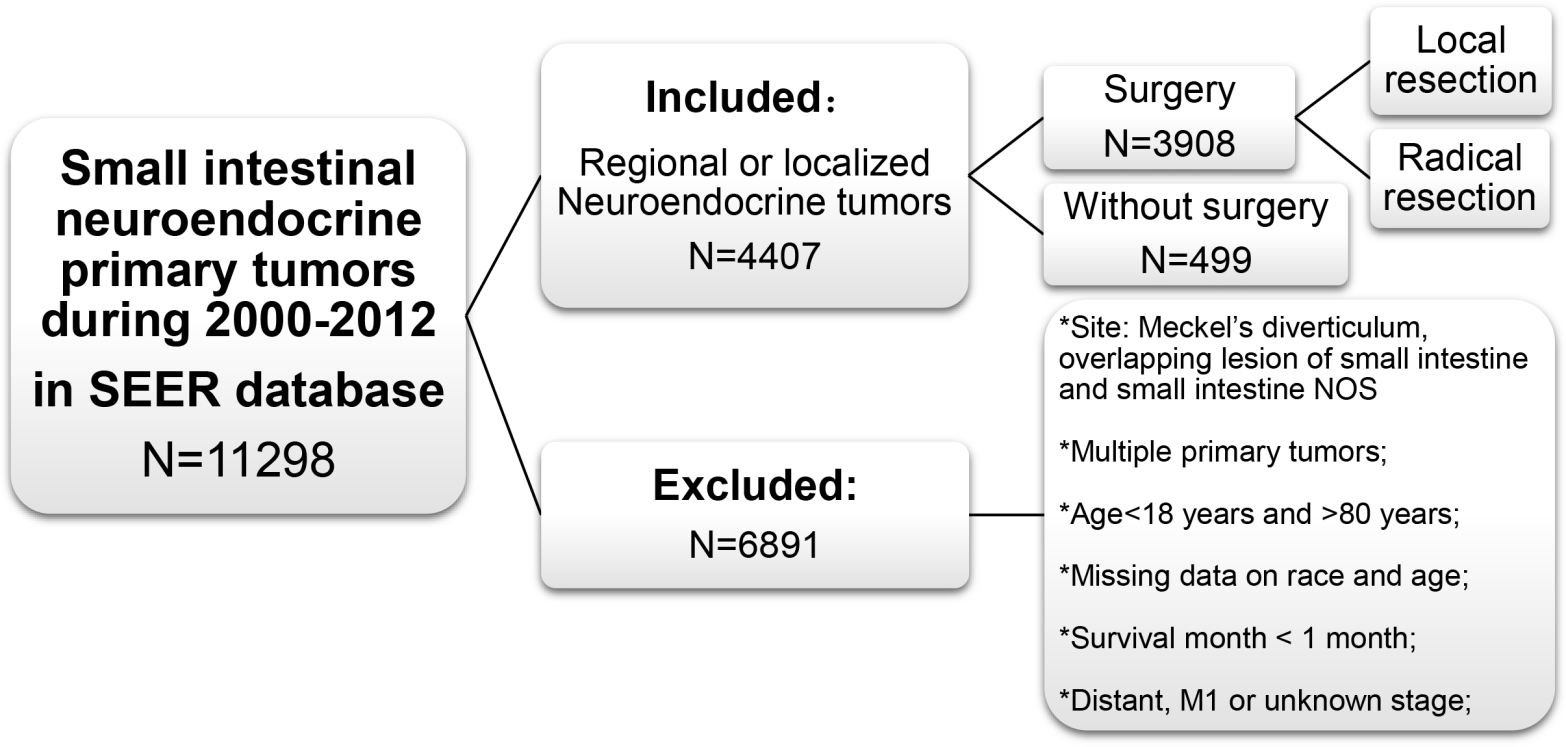
Cohort inclusion and exclusion diagram NOS: not otherwise specified.

The exclusion criteria were as follows: (1) patients lacking documentation of race or age at diagnosis; patients younger than 18 years or older than 80 years; (2) patients with multiple primary tumors; (3) patients surviving less than one month because they may die of surgical complications or rapidly progress after palliative resection; (4) patients with distant metastatic disease (M1). (5) Patients diagnosed with tumors within a Meckel's diverticulum or overlapping lesion of small intestine (Figure 6).

### Identification of surgery codes

Type of operation was defined by site-specific surgery codes provided by SEER. Local resection (LR) was defined as simple resection, polypectomy, and excisional biopsy, excision of lesion, simple removal of lesion, or partial removal of lesion. Radical resection (RR) was defined as debulking or radical resection with an en bloc resection (partial or total removal) of other organs [41].

### Statistical analysis

All cases were regrouped according to the 7^th^ AJCC TNM [8, 40] and 2006-20007 ENETS [42, 43] stage system, for comprehensive and comparable reporting. Site of tumor was coded as duodenum, jejunum and ileum. Race was divided into white, black and others. Age was classified into young (≤50 years old) and old (>50 years old) groups. Histology was classified into carcinoid tumor (8240/9249), neuroendocrine cancer (8246/8013/8041), and other types (small sample sizes of other subtypes except carcinoid and neuroendocrine). Descriptive statistics for variables are reported as count with percentage or mean with interquartile range. Age-adjusted incidence rates were calculated using SEER. Survival curves were generated using Kaplan-Meier methods, and the log-rank test was carried out to evaluate the survival differences between groups. Cancer-specific survival was calculated from the date of diagnosis to the date of death from cancer. Death attributed to other causes was defined as a censored observation. Adjusted hazard ratios along with 95% confidential intervals (CI) were calculated using Cox proportional hazards regression model. T-stage was considered as ordered categorical covariates, whereas race and histological type were considered as non-ordered categorical covariates. Differentiated grade was divided into well differentiated and moderately/poor/undifferentiated group. Stratified analysis of surgical management was performed in age, race, gender, location, tumor size, differentiate grade and T/N stage. We used 8 lymph nodes as a cutoff value because resection of >8 LNs was associated with a better survival in patients with SiNETs. [24] Missing values in numerical variables were transformed by the method of mean of other points and missing values less than 10% in categorical variables were transformed by the method of maximum relative frequency. [44] When the two-side *P* value was less than 0.05, the difference was considered statistically significant. SPSS 22.0 (SPSS Chicago IL. USA) software was used for data analysis.

## CONCLUSIONS

Early SiNETs have a favorable prognosis. Surgical resection may improve outcomes, particularly in older patients and those with large tumors. More aggressive resections couldn't improve outcomes.

## SUPPLEMENTARY FIGURE



## Abbreviations

SiNETs: Small intestinal neuroendocrine tumors
NETs: Neuroendocrine tumors
LNs: Lymph nodes
SEER: Surveillance, Epidemiology and End Results
AJCC: American Joint Committee on Cancer
WHO: World Health Organization
CSS: Cancer-specific survival
HR: Hazard ratio
CI: Confidence interval
IQR: Interquartile range
NOS: Not otherwise specified
LR: Local resection
RR: Radical resection.
