# Supplementary material for: Survival outcomes and surgical intervention of small intestinal neuroendocrine tumors: a population based retrospective study
Source: Oncotarget. 2016 Nov 26;8(3):4935–47. doi: 10.18632/oncotarget.13632 (PMC5354882; doi:10.18632/oncotarget.13632)
Supplement: Supplementary file 1 [file oncotarget-08-4935-s001.pdf]

Survival outcomes and surgical intervention of small intestinal neuroendocrine tumors: a population based retrospective study

SUPPLEMENTARY FIGURE

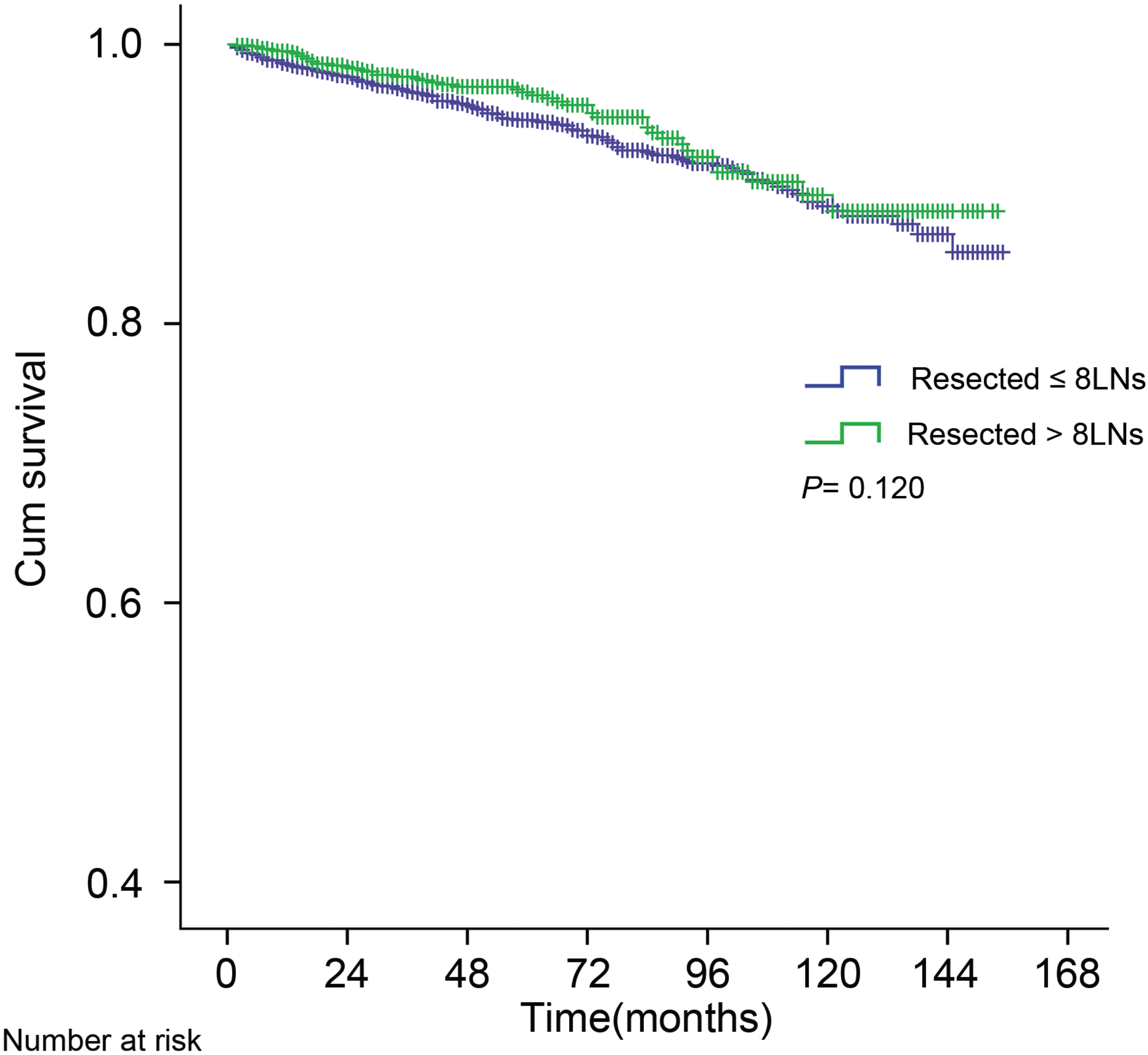

Number at risk

	0	24	48	72	96	120	144	168
LNs ≤8	2996	2224	1548	1010	571	274	71	0
LNs >8	1351	926	589	344	185	77	16	0

Supplementary Figure S1: The cancer-specific survival curve according to the number of lymph node removed in localized SiNETs patients. LNs of 8 was used as cutoff value. *P* value from log-rank test. LNs, lymph nodes.
